# Atomic Layer Deposition of Ni-Co-O Thin-Film Electrodes for Solid-State LIBs and the Influence of Chemical Composition on Overcapacity

**DOI:** 10.3390/nano11040907

**Published:** 2021-04-02

**Authors:** Yury Koshtyal, Ilya Mitrofanov, Denis Nazarov, Oleg Medvedev, Artem Kim, Ilya Ezhov, Aleksander Rumyantsev, Anatoly Popovich, Maxim Yu. Maximov

**Affiliations:** 1 Peter the Great Saint-Petersburg Polytechnic University, 195221 Saint Petersburg, Russia; yury.koshtyal@gmail.com (Y.K.); carlemeros@gmail.com (I.M.); dennazar1@ya.ru (D.N.); medvedev.os1990@gmail.com (O.M.); artem_7.kim@mail.ru (A.K.); iezhov1994@gmail.com (I.E.); rumyantsev.amr@gmail.com (A.R.); director@immet.spbstu.ru (A.P.); 2 Saint Petersburg State University, 199034 Saint Petersburg, Russia; 3 Ioffe Institute, 194021 Saint Petersburg, Russia

**Keywords:** atomic layer deposition, nickel–cobalt oxide, anode materials, solid–state Li-ion batteries, overcapacity, SEI

## Abstract

Nanostructured metal oxides (MOs) demonstrate good electrochemical properties and are regarded as promising anode materials for high-performance lithium-ion batteries (LIBs). The capacity of nickel-cobalt oxides-based materials is among the highest for binary transition metals oxide (TMOs). In the present paper, we report the investigation of Ni-Co-O (NCO) thin films obtained by atomic layer deposition (ALD) using nickel and cobalt metallocenes in a combination with oxygen plasma. The formation of NCO films with different ratios of Ni and Co was provided by ALD cycles leading to the formation of nickel oxide (a) and cobalt oxide (b) in one supercycle (linear combination of a and b cycles). The film thickness was set by the number of supercycles. The synthesized films had a uniform chemical composition over the depth with an admixture of metallic nickel and carbon up to 4 at.%. All samples were characterized by a single NixCo1-xO phase with a cubic face-centered lattice and a uniform density. The surface of the NCO films was uniform, with rare inclusions of nanoparticles 15–30 nm in diameter. The growth rates of all films on steel were higher than those on silicon substrates, and this difference increased with increasing cobalt concentration in the films. In this paper, we propose a method for processing cyclic voltammetry curves for revealing the influence of individual components (nickel oxide, cobalt oxide and solid electrolyte interface—SEI) on the electrochemical capacity. The initial capacity of NCO films was augmented with an increase of nickel oxide content.

## 1. Introduction

Small-sized power sources, with large cyclic capability and high energy density, such as solid-state lithium-ion batteries (SSLIB), are in demand owing to rapidly developing technologies including the Internet of Things (IoT). The IoT concept involves the use of a large number of wireless miniature devices (sensors [1], activators, real-time clocks [2], etc.), which require the implementation of the energy harvesting principle [3] to ensure their autonomous operation.

SSLIBs offer the following advantages over lithium-ion batteries (LIBs) with liquid electrolytes: increased fire safety, long-term static and dynamic stability without self-discharge and long service life [4,5], unlike LIBs with liquid electrolytes. In addition, in SSLIBs it is possible to increase the charge voltage and the voltage of the battery itself, making it possible to increase the energy density [4,5].

Binary transition metal oxides (TMOs), owing to their high capacity (e.g., ZnFeO_2_—700 mAh/g, ZnCo_2_O_4_—867 mAh/g, NiCoO_2_—716 mAh/g, NiCo_2_O_4_—891 mAh/g), are very promising materials for producing SSLIB anodes. However, low conductivity and relatively high-volume change during cycling are the main challenges limiting their use [6,7]. To overcome these challenges, it is necessary to utilize various nanostructures that are more stable with repeated cycling than bulk materials of anodes [6,8,9]. Moreover, 2-D nanostructures, called nanofilms, can be used for producing thin-film SSLIBs to minimize the size of electronic devices.

Thin-film components (cathodes, anodes, solid electrolytes) can be manufactured using atomic layer deposition (ALD), chemical vapor deposition (CVD), pulsed laser deposition (PLD), magnetron sputtering, and others [10,11,12]. Among these methods, ALD is the most promising since it allows the synthesis of uniform and conformal films of various materials on flat and 3-D substrates of complex shapes [13,14]. In this case, it is possible to control the thickness of the deposited coatings with high precision.

Numerous studies have addressed the preparation of individual components of thin-film SSLIBs by ALD, such as cathodes [15,16,17,18,19,20,21,22,23,24,25,26], anodes [27,28,29,30,31,32,33,34,35,36,37,38,39,40,41,42,43,44,45,46,47,48,49] and solid-state electrolytes [50,51,52,53]. Using several precursors in the ALD chamber allows the fabrication of all the SSLIB components using ALD equipment [12]. However, thin-film materials based on binary TMOs of nickel and cobalt (NiCo_2_O_4_ and NiCoO_2_) fabricated by ALD [54] have not been studied as LIB anodes.

The aim of this work was to prepare thin-film anodes of binary TMOs of nickel and cobalt by ALD using different ratios of pulses of metal-containing precursors. In addition to studying the composition, morphology and crystallinity of the deposited films, special attention was paid to characterizing the electrochemical properties.

Previously, we showed an increase in the electrochemical activity of NiO anodes caused by an increase in the electrochemical activity of the SEI (solid electrolyte interphase) films [55]. In this regard, the method for determining the capacity in disk cells using an electrolyte may be inaccurate due to the SEI contribution to the measured capacity. In addition, the growth rate of SEI films can depend on the nature of the anode material, which introduces additional uncertainty in the characteristics of the films under study. Fabricating multilayer structures of solid-state thin-film LIBs is a difficult task and the determining characteristics of active materials (anodes) can be limited by other system components such as the solid electrolyte and cathode. Therefore, within the framework of the described research objectives, an attempt was made to separate the various components in the results of electrochemical testing in a liquid electrolyte.

## 2. Materials and Methods

Monocrystalline silicon substrates (surface orientation (100), 40 × 40 mm, Telecom-STV Co., Ltd., Zelenograd, Russia) and stainless-steel plates (316SS, 16 mm diameter, Tob New Energy Technology Co., Ltd., Xiamen, China) were used as substrates for ALD. Prior to deposition, the silicon and stainless-steel substrates were cleaned in an ultrasonic bath in acetone and deionized water for 10 and 5 min, respectively. To remove the native silicon oxide layer, the silicon substrates were immersed for 5 min in 10% hydrofluoric acid solution(HF). Then, silicon substrates were cleaned using piranha solution (H_2_SO_4_/H_2_O_2_, volume ratio 7:3) for 20 min to remove organic contaminants and produce a hydroxylic surface. Finally, the silicon substrates were rinsed in deionized (DI) water and dried under an argon atmosphere [29].

ALD of nickel–cobalt oxide (NCO), nickel oxide (NO), and cobalt oxide (CO) were performed with a commercial R-150 setup (Picosun Oy, Espoo, Finland) at a temperature of 300 °C and a reactor base pressure of 8–12 hPa. Bis(cyclopentadienyl) nickel(II) and Bis(cyclopentadienyl) cobalt(II) (Ni(Cp)_2_, Co(Cp)_2_; 99%, Dalchem, Nizhny Novgorod, Russia) were used as the nickel and cobalt-containing precursors. Co(Cp)_2_ and Ni(Cp)_2_ were kept in stainless-steel bottles (Picohot™ 200, Picosun Oy) and heated during deposition to 160 and 110 °C, respectively. The pulse times and purge times were 1 s and 10 s for both Co(Cp)_2_ and Ni(Cp)_2_. Remote oxygen plasma was used as a counter-reagent. The plasma power was 3 kW, with a frequency range of 1.9–3.2 MHz. The total plasma pulse time was 19.5 s (Ar purge during 0.5 s with flow rate 40 sccm; Ar and O_2_ plasma purge during 14 s with flow rate 90 sccm; Ar purge during 5 s with flow rate 40 sccm). Deposition conditions were based on our previous studies devoted to obtaining cobalt and nickel oxide [55,56].

The spectroscopic ellipsometry parameters (Ψ and Δ) for films deposited on a silicon substrate were measured out with an Ellips-1891 SAG ellipsometer (CNT, Novosibirsk, Russia) in a wavelength range from 370 to 1000 nm and an incidence angle of 70°. The Spectr software package (1.10, CNT, Novosibirsk, Russia) was used to construct and fit a structural-optical model function. After fitting the parameters of the optical model and experimental spectra, the thicknesses of the films were calculated. The errors of the film thickness calculation were no more than 0.3 nm. The gradient of the thickness (*GT*) was calculated using Equation (1):

(1) $$GT=\frac{T_{\max}-T_{\min}}{T_{\max}+T_{\min}}\times 100\%,$$

where T_max_ and T_min_ are the maximum and minimum film thicknesses, respectively [29].

X-ray reflectometry (XRR) and X-ray diffraction (XRD) studies were performed with a Bruker D8 DISCOVER (Cu-Kα = 1.5406 Å) diffractometer. Surface-sensitive grazing incidence XRD (GIXRD) modes were used for XRD measurements using a 2θ range of 30–65° with a step of 0.1° and an exposure of 1 s at each step. The incidence angle of the primary X-ray beam was 0.7°. XRR measurements were performed in an angle range of 0.3–5° (increment 0.01°) using symmetric scattering geometry. The obtained results were processed by the Rietveld method using the TOPAS software package (ver. 5, Bruker, Billerica, MA, USA) and by the simplex method using the LEPTOS (ver. 7.7, Bruker, Billerica, MA, USA) for XRD and XRR, respectively.

X-ray photoelectron spectra (XPS) were acquired with an Escalab 250Xi spectrometer (Thermo Fisher Scientific, Waltham, MA, USA). The samples were sputtered by Ar^+^ ions with an energy of 500 eV for 30 and 45 or 90 s. The samples were excited by Al-Kα (1486.7 eV) X-rays at a pressure of 7 × 10^−8^ Pa.

Scanning electron micrographs of planar and cross-sectional views were obtained by a Supra 55 VP scanning electron microscope (SEM, Zeiss, Oberkochen, Germany) with a Gemini-I column and a field emission cathode. Spatial resolution was about 1.3 nm at an accelerating voltage of 15 kV. A total of 3–4 randomly selected positions on the surface of the sample were investigated. Everhart–Thornley and InLens secondary electron detectors were used for SEM studies. Energy-dispersive X-ray (EDX) analysis was performed using the INCA X-Max system (Oxford Instruments, High Wycombe, UK) installed on the SEM Supra 55 VP.

Stainless-steel plates with deposited films were used for electrochemical studies. Lithium foil, polyolefin porous film 2325 (Celgard, Charlotte, NC, USA), and TC-E918 (Tinci, Guangzhou, China) solutions were used as the counter, separator, and electrolyte, respectively. The composition of TC-E918 was a 1M solution of LiPF_6_ in a mixture of organic carbonates (ethylene carbonate, propylene carbonate, diethyl carbonate, ethyl methyl carbonate, vinylene carbonate). The coin cells (CR2032) were assembled in an argon atmosphere using an OMNI-LAB glove box (VAC). Cyclic voltammetry (CV) was performed using a PGSTAT302N+ potentiostat (Autolab, Utrecht, The Netherlands) in a range of 0.01–3.00 V with a scan rate of 0.5 mV/s. Discharge tests at different current densities were run with a CT3008W-5V10mA charge/discharge stand (Neware, Shenzhen, China), calibrated to work with low currents in a voltage range from 3.00 V–0.01 V, and current densities of 10, 20, 40, 80, 160, 320, 480, 640, 800 µA/cm^2^. Deconvolution of the CV patterns was performed using the Origin (ver. 9.0.0) software package.

## 3. Results and Discussion

### 3.1. Atomic Layer Deposition of Ni-Co-O Thin Films

First, we determined the conditions for ALD of oxide systems (NiO, CoO) based on previous experience [55,57], as shown in Table 1. The optimal temperature of the reactor was 300 °C, and for stainless-steel bottles with NiCp_2_ and CoCp_2,_ the optimal temperatures were 110 and 150 °C, respectively. The pulse times of the metal-containing reagent(A) and oxygen plasma(B) were 1 and 15 s, respectively, for both processes.

To prepare binary TMOs using ALD, we used the supercycle approach. This approach takes the common binary ALD cycles of precursor and coreactant pulses for each constituent process and combines them into a cycle of cycles [58]. In the NiCoO_2_ phase, the ratio Ni/Co = 1; therefore, we used the ratio of the cycles of obtaining NiO and CoO in one supercycle, which is also equal to 1. However, given that growth per cycle (GPC) for CoO (0.22 Å) was almost twice the increase per cycle for NiO (0.12 Å), we also synthesized samples with a large number of NiO cycles in one supercycle (Table 2).

The thicknesses of the films on silicon substrates were measured using spectroscopic ellipsometry and XRR. The XRR curves are represented in supplement materials (Figures S1–S5). The growth per cycle (GPC) and per supercycle (GPSC) are presented in Table 2. The XRR data showed slightly lower values than ellipsometry data. Based on the GPC values obtained from ellipsometry for pure NiO and CoO, the thicknesses, GPC, and GPSC were calculated for the binary TMO samples (Table 2). These calculations assumed that the GPC of NiO and CoO remained constant when going from standard cycles to supercycles. The calculated values turned out to be 10–20% less than the experimental ones, which indicates the stimulation of the growth of one or both phases on the surface of the other when using supercycles.

### 3.2. Chemical Composition of the Films

XPS and SEM-EDX were used to determine the chemical composition of the samples. Measurements of XPS spectra were made after sputtering of the surface layer using the argon ions (3 keV). To determine homogeneity of a chemical composition, different sputtering times were used (0, 30 and 45 or 90 s). Results of XPS without sputtering showed high carbon content (>20%) due to adventation carbon contamination. After sputtering, the carbon content decreased remarkably to 2–4% depending on the type of sample (Table 3).

**Figure 1:**
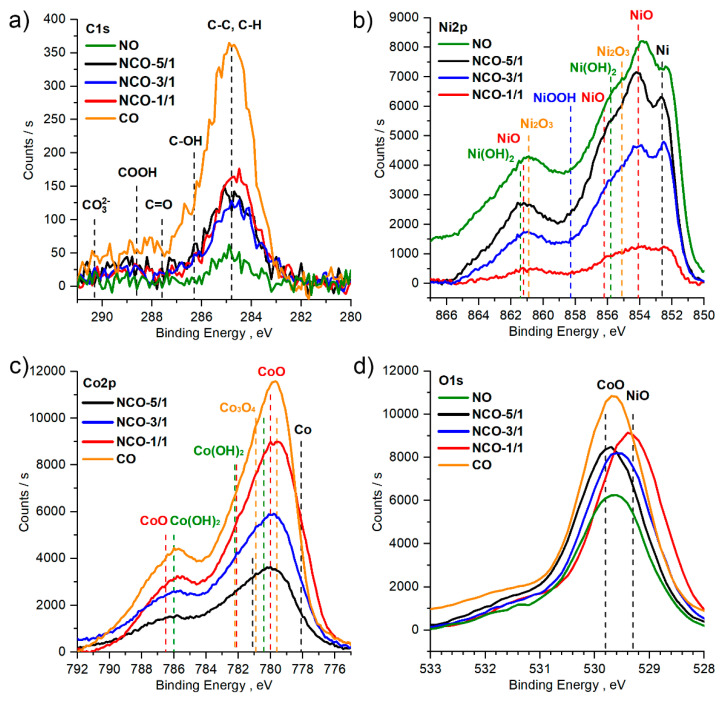
X-ray photoelectron spectra after Ar+ ion-sputtering during 90 s for NO, NCO and 45 s for CO: (**a**) C1s, (**b**) O1s, (**c**) Ni2p, (**d**) Co2p.

In the C1s spectra (Figure 1a), for all samples and regardless of the sputtering time, only one peak (maxima at 284.8 eV) was clearly visible, corresponding to the carbon of the C-C and C-H. The carbon was likely from cyclopentadienyl nickel and cobalt, which can thermally decompose at the temperatures required for ALD [59]. In general, the peak with a maximum at 284.8 eV was symmetric. No additional peaks in the range of 287–292 eV, corresponding to carbon found in various oxygen-containing groups (hydroxyl, aldehyde, carboxyl etc.) were observed.

The spectrum for Ni2p (Figure 1b) was complex because of the significant overlap of the Ni3d and O2p orbitals, resulting in broad peaks with multiple satellite peaks [59,60,61]. However, the shape and position of the maxima showed that all the samples under study contain NiO (854.1 eV) and Ni^+3^ (Ni_2_O_3_ and/or Ni_3_O_4_), or nickel hydroxide—Ni(OH)_2_. For all samples, a peak in the region with a maximum of 852.7 eV, corresponding to metallic nickel, was clearly visible. The formation of metallic nickel is caused by the decomposition of the initial precursor, a cyclopentadienyl nickel. This was observed in a number of other studies, including CVD using Ni(C_5_H_5_)_2_ and O_2_ as precursors [62].

The Co2p spectrum was also quite complex (Figure 1c), as it contained a combination of many components, and could not be decomposed into components with high accuracy and reliability. However, according to the position of the Co2p maxima both in the NCO series and in the CO sample, cobalt existed predominantly in the form of CoO. However, the presence of metallic cobalt and/or Co_3_O_4_ in small amounts was also possible.

The oxygen content in the films varied between 41–47% and was slightly less than the total content of cobalt and nickel due to the presence of metallic nickel and possibly cobalt. The shape of the O1s spectra was similar for all samples (Figure 1d). The positions of the maxima for the samples NO (NiO), 529.6 eV, and CO (CoO), 529.7 eV, were nearly identical, are in agreement with the literature data [63,64]. However, a shift of the peaks towards lower energies was observed with decreasing nickel concentration, although the shift was small and the maxima of all peaks were in the range of 529.4–529.7 eV. Such small displacements can be caused by inaccuracies in charge compensation during the normalization of the spectra to the C1s line, which was very noisy for these samples (Figure 1a).

It should also be noted that the content of elements and the shape of the corresponding spectra C1s, O1s, Ni2p, Co2p for different sputtering times did not differ significantly, indicating the homogeneity of the composition of the films over depth of the film.

### 3.3. Crystal Structure

**Figure 2:**
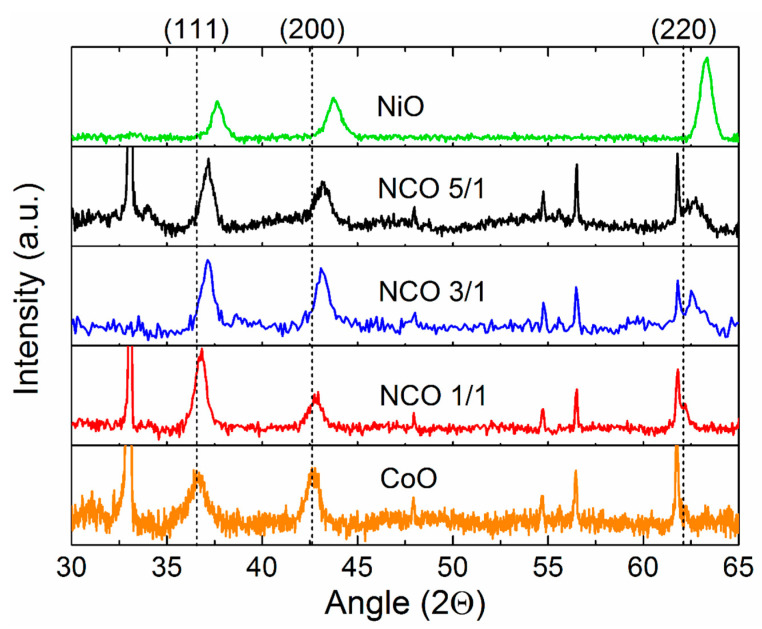
The X-ray diffraction patterns of the CoO, NCO-1/1, NCO-3/1, NCO-5/1, NiO thin films deposited on Si. The dashed lines show the CoO peak positions.

Figure 2 shows the diffraction patterns of the samples obtained by the grazing-incidence method. The broad diffraction peaks in the region of 37°, 43°, and 63° were characterized by a cubic structure with the space group Fm-3m. Narrow peaks at 33° and in the range of 54–62° were associated with defects in the crystal structure of silicon substrates. The absence of peaks at 31° and 56° indicated a gradual substitution of cobalt atoms for nickel atoms without the formation of intermediate oxide forms of spinel (Ni_x_Co_1−x_)_3_O_4_ (PDF 00-042-1467), which were observed in [54]. Thus, the phase composition of the samples was characterized by a single Ni_x_Co_1−x_O phase with a cubic face-centered lattice. The unit cell parameter refined by the Rietveld method for all samples is presented in Table 4.

It can be seen from the results of X-ray diffraction analysis that with an increase in the Ni content in the Ni_x_Co_1−x_O phase, the unit cell parameter decreased from 4.244 to 4.161 Å, which was reflected in a smooth shift of the positions of the peaks (111), (200), and (220) (in the XRD patterns, the dashed lines show CoO peak positions). The unit cell parameter of NiO and CoO films closely agreed with the data for compact materials NiO (4.16 Å) and CoO (4.24 Å). As expected it increased with an increase in the proportion of CoO compared to NiO. From the ratio of the intensities of the peaks, a texture in the (111) direction was observed in the samples, which is typical for structures with a cubic system. The absence of reflections of metallic nickel, the presence of which was determined using XPS, may indicate its low fraction in the volume of the film or X-ray amorphousness due to the small size of crystallites.

According to XRR data (Table 4) obtained by the simplex method, all samples consisted of a uniform density layer with a roughness of 0.63 to 3.23 nm. As the number of NiCp_2_ pulses in the supercycle decreased, the film density also decreased from 6.60 to 6.31 g/cm^3^ (except for the NCO-1/1 sample), which was caused by a relative increase in the amount of the less dense CoO phase (6.72 g/cm^3^ for NiO and 6.44 g/cm^3^ for CoO). The low density of thin films in comparison with compact oxides NiO and CoO was associated with a large influence of surface effects [55].

### 3.4. Morphology of Films on Si and Steel Substrates

**Figure 3:**
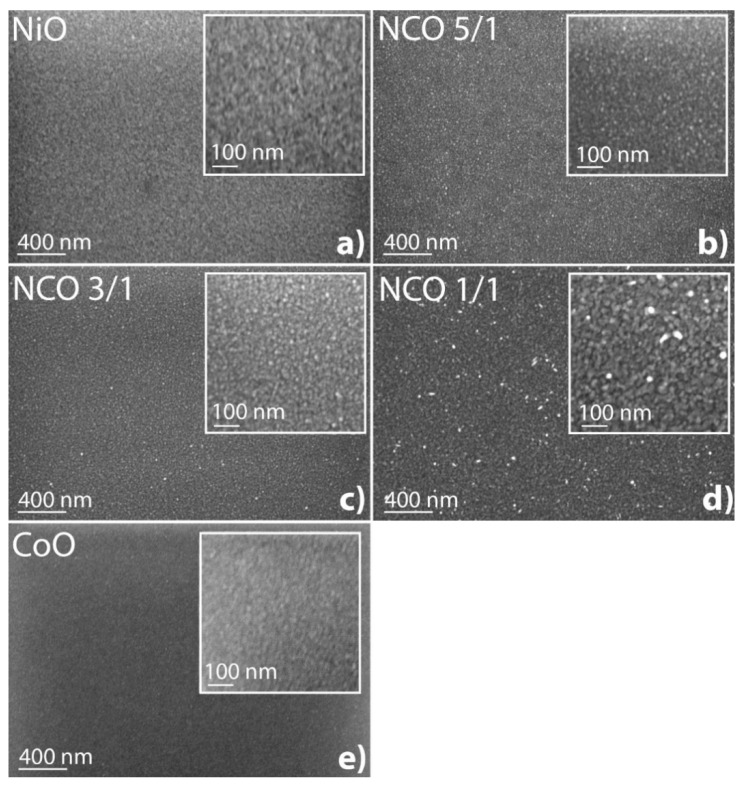
Scanning electron microscopy (SEM) images of (**a**) NiO, (**b**) NCO-5/1, (**c**) NCO-3/1, (**d**) NCO-1/1 and (**e**) CoO. thin films deposited on silicon.

Scanning electron microscopy images of NiO, NCO-5/1, NCO-3/1, NCO-1/1, and CoO films on Si substrate are presented in Figure 3. For the monoxide films (NiO and CoO), the surfaces were homogeneous and free of inclusions. Bright inclusions with a diameter of 10–13 nm (NCO-5/1, NCO-3/1) and 25–30 nm (NCO-1/1) were visible on the NCO film samples, as roughness increased in the series NCO-5/1—NCO-3/1—NCO-1/1, which is consistent with the XRR results (Table 4). It should be noted that the bright features of the NCO-1/1 sample were randomly directed nanowire crystals on the surface, the growth features of which will be discussed below.

The XPS results show that NCO films contained metallic nickel and, possibly, metallic cobalt as a result of the decomposition of precursors NiCp_2_ and CoCp_2_ [62]. It can be assumed that bright inclusions comparable with the film thickness were metallic particles of nickel and cobalt, a higher density of which gives an increased yield of secondary electrons that increases their contrast in comparison with the film.

**Figure 4:**
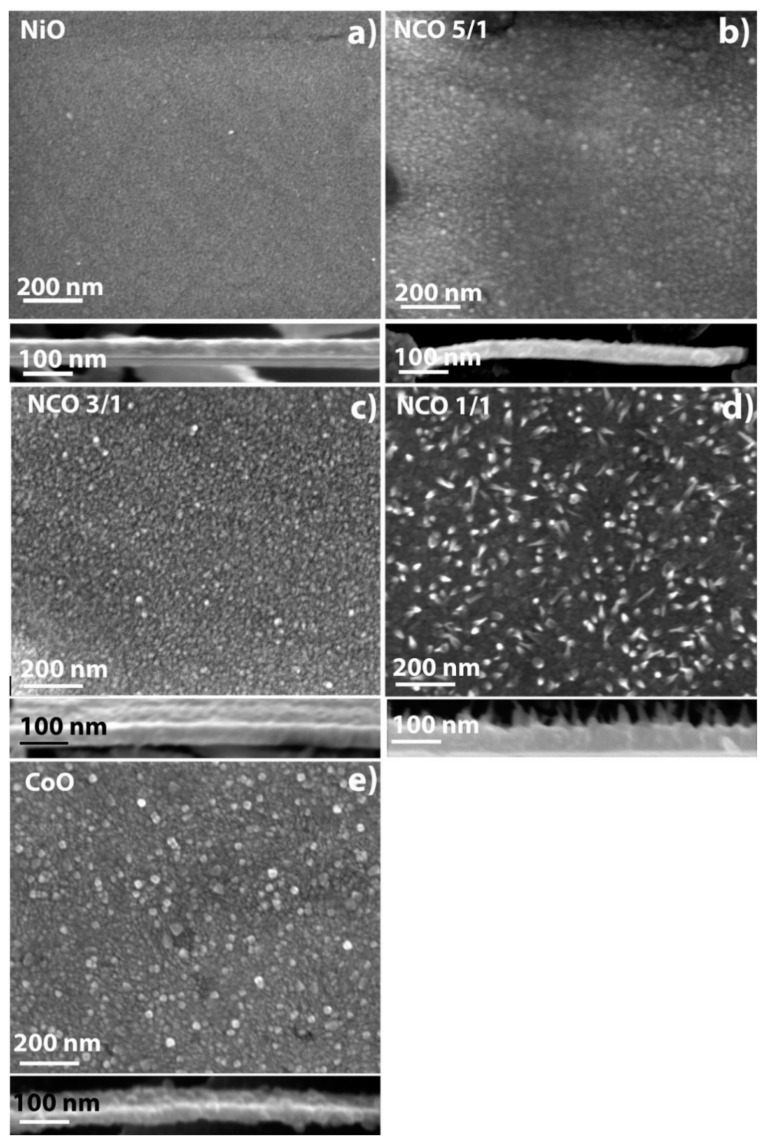
Scanning electron microscopy of plan view and cross section of the thin films deposited on steel: (**a**) NiO, (**b**) NCO-5/1, (**c**) NCO-3/1, (**d**) NCO-1/1 and (**e**) CoO.

Plan view and cross-section images of the studied films on stainless a steel substrate are shown in Figure 4. The general features of the film morphologies of silicon and steel were similar. As in the case of films on silicon, the growth rate increased with the cobalt content, though it should be noted that the thickness of the films on steel with simultaneous deposition process was greater, which indicates an increased growth rate of films on a steel substrate (see Table 5). Table 5 also presents the typical sizes of surface inclusions observed in NCO films on silicon (spectral ellipsometry results) and steel (SEM results).

It should be noted that, as in the case of NCO-1/1 on silicon, the film on steel was covered with randomly directed nanowire crystals, but their number was noticeably higher, which is most likely associated with a large number of crystallization centers and inhomogeneities on the steel substrate (due to the increased roughness) rather than silicon. On the cross-section view of the NCO-1/1 sample (Figure 4d bottom), nanowire crystals can be easily observed, reaching lengths up to 40 nm. Several mechanisms of the nanowire/whiskers growth are presented in the literature, describing the morphology of the sample NCO-1/1 [65,66,67], including the growth of metal-oxide whiskers [68,69,70]. During the formation of TMO nanowires, the importance of the chemical composition and growth conditions were noted [69,70]. Most likely, in the case of sample NCO-1/1, these two conditions were fulfilled, stimulating the growth of nanowire crystals on the surface. A more detailed discussion is presented in the Supplementary Materials.

SEM images of the CoO sample are presented in Figure 4e. The surface of the CoO film on the steel substrate was represented by large crystallites with diameters of 25–35 nm, and small crystallites with diameters of 10–15 nm, similar to the features on the NCO-5/1 and NCO-3/1 samples.

### 3.5. Electrochemical Studies

**Figure 5:**
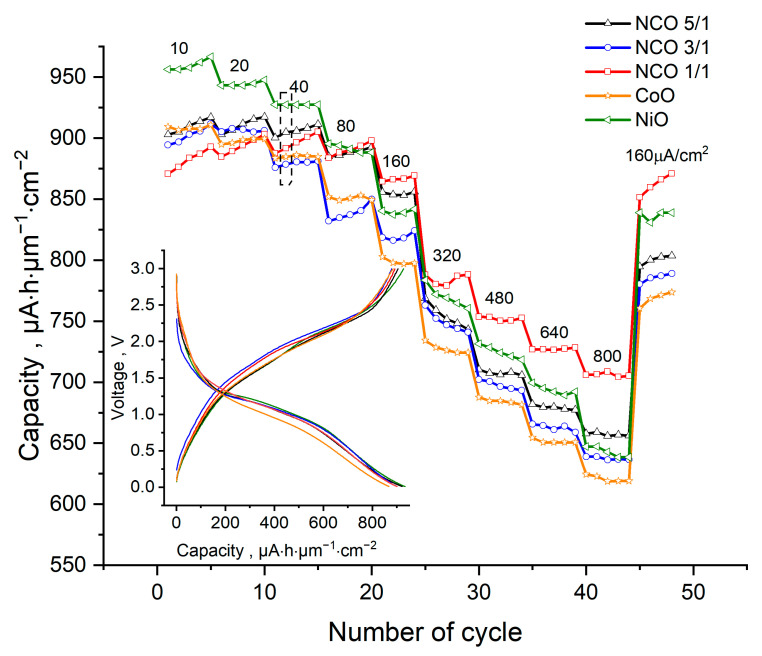
Rate performance and charge/discharge profile of the thin films. Charge/discharge curves are represented as dots in the dotted frame.

Lithium metallic foil was used as a counter electrode during electrochemical studies of samples deposited on stainless steel. The current density varied from 10 µA/cm^2^ (~0.3C) to 800 µA/cm^2^ (~33C) (Figure 5). It was found that the charging capacities of investigated samples were stable during cycling at a given charge current density. The inset of Figure 5 shows typical charge-discharge curves at a current density of 40 µA/cm^2^ (approximately 1C) in the potential range from 0 to 3 V. The main contribution to the capacity during charging/discharging occurred at potentials of 1.5–2.2 V/1.5–0.75 V.

According to XPS and XRD measurements, CoO, NiO, and NiCoO_2_ (Fm-3m) phases were present in the deposited films, while spinel crystal phases of Co_3_O_4_ and Ni_x_Co_(1−x)3_O_4_ were not. We calculated the specific capacities per unit mass and volume based on the assumed electrochemical processes occurring with detected phases (Equations (2)–(4)) and experimental data [7,71,72,73]. The results are summarized in Table 6.

NiCoO_2_ + 4Li^+^ + 4e^−^ → Ni + Co + 2Li_2_O, (2)

NiO + 2Li^+^ + 2e^−^ → Ni + Li_2_O, (3)

CoO + 2Li^+^ + 2e^−^ → Co + Li_2_O, (4)

As the discharge current increased from 0.3C to 33C, the charge capacities decreased from 909–967 µAh/cm^2^/µm (1441–1460 mAh/g) to 625–706 µAh/cm^2^/µm (990–1140 mAh/g). Such a moderate decrease in capacity (21–33%), with a significant increment of current density, was apparently due to the small thickness of the electrochemically active films.

The measured capacities of thin-film electrodes were higher than the theoretical values and those found in the literature. Greater values for measured capacities over theoretical values for samples with similar chemical composition were also observed previously [7,71,72,73]. The difference between measured and theoretical capacities was likely caused by the additional capacity of an organic polymer film formed on the surface of the active material as a result of reactions between Ni and Co metal and electrolyte at electrode potentials below 0.4 V [7].

At fixed current densities above 160 μA/cm^2^ (~5C), the charge capacities were augmented with additional nickel content in the deposited film. An exception to this trend was the NCO-1/1 sample, which exhibited the highest specific capacity (870–706 µAh/cm^2^/µm)) at current densities of 160–800 μA/cm^2^. The observed superior capacity of the NCO-1/1 sample was likely due to the formation of whiskers during depositions, which have higher surface areas (Figure 4. SEM of steel).

**Figure 6:**
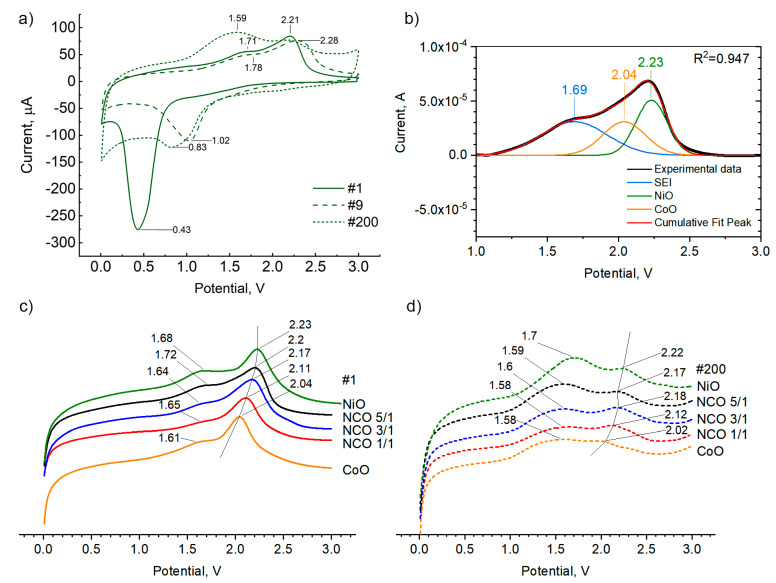
Cyclic voltammetry curves of the NCO-5/1 sample. (**a**) 1, 9 and 200 cycles, (**b**) example of deconvolution of the cyclic voltammetry (CV) curve of the NCO-5/1 sample, (**c**) anode CV curves of the films before, (**d**) after cycling (after 200 cycles) related to the thickness of the substrate. Presented curves for (**c**,**d**) plotted in the same coordinate system but physically spaced.

**Figure 7:**
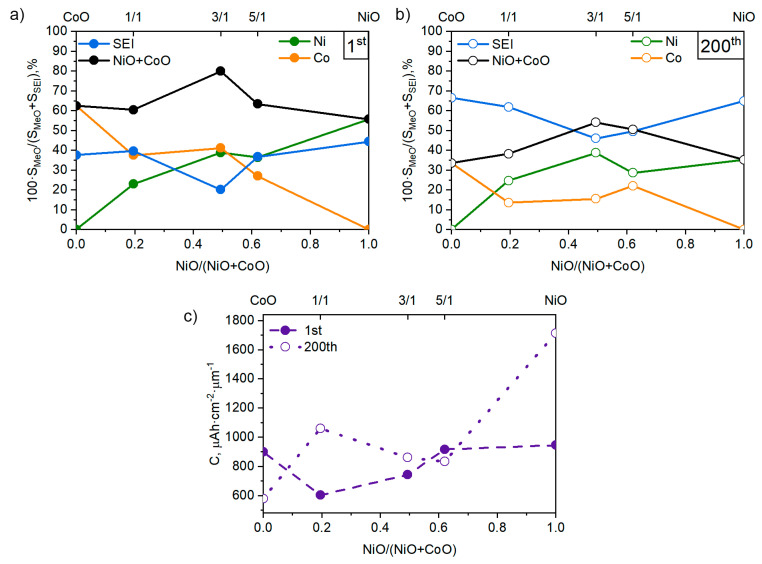
Components’ contribution (**a**) before cycling, (**b**) after cycling, and (**c**) charge capacities of the films.

Cyclic charge/discharge of electrodes resulted in a gradual increase in the discharge capacity. Wei et al. [72] (Figure 7c, AT curve) also observed capacity growth during cycling of active material with a similar chemical composition (NiO-CoO/carbon fiber). However, a detailed study of this phenomenon has not been found. The increase in capacity during cycling is likely directly related to the growth of SEI films and may depend on the nature of the anode material and the electrolyte used. Consequently, in the study of thin films’ electrochemical properties, the contribution from SEI cannot be neglected, in contrast to the study of bulk anode/cathode materials. Considering the results of our previous studies on NiO films [55], cyclic voltammetry was applied to identify electrochemical processes occurring during the charging/discharging of nickel-cobalt mixed oxide films (Figure 6).

At the beginning of the CV study, the open circuit voltage was close to 3 V. As the electrode potential gradually decreased, the conversion reaction was more likely to occur [74]. Furthermore, in the cathode area (0.8–0.1 V, with a maximum at 0.43 V), a dramatic current increase was observed. A similar rise in current was observed when carbon materials were studied using electrolyte, signifying the reduction of the electrolyte components. With a subsequent increase in potential from 0 to 3 V in the anodic region, current peaks of 1.6–1.7 V and 2.0–2.3 V were observed, characterizing the changes of SEI film and transition metal oxides (NiO and CoO), respectively (Figure 6a,c).

To understand the origin of current increment at 1.6–1.7 V of the anode curve, the effect of the potential width range on the form of the CV curve was studied using NCO-5/1 samples. For the first cycle, when the potential range was narrowed to 3.0–0.8 V, the current increase of 1.6–1.7 V potential range of the cathode curve did not appear (Figure S6). Thus, the augmentation of current in the 0.8–0.1 V region (cathode curve) and in 1.6–1.7 V (anode curve) is connected and may be due to SEI film formation, which can significantly contribute to the reversible capacity. The transferred charge in 0.8–3.0 V and 0.1–3.0 V was 0.0709 C (conversion reaction at 0.8–3.0 V) and 0.321 C (conversion reaction in 0.01–3.0 V range and SEI formation 0.01–0.8 V). When cycling from 0.01 V to 3.0 V (first to ninth cycle), the cathode peak observed at 0.43 V (first cycle) shifted towards higher potentials up to 1.0 V, signifying the transformation of the SEI film.

Performing 200 charge/discharge cycles in the 0.01–3.0 V potential range of the NCO-5/1 sample resulted in a shift of the maximum from 1.6–1.7 V region towards lower potentials and an increase its intensity. Furthermore, specific capacity increased from 768 to 833 μAh/cm^2^/µm from 25 to 200 cycles, respectively (measured at 320 µA/cm^2^ for NCO-5/1 (Figure 6c,d).

After conducting 5 and 200 charge/discharge cycles, the NCO-5/1 electrodes were taken from CR2032 cases, then rinsed thoroughly with dimethyl carbonate (DMC) and investigated by scanning electron microscopy (Figure S7). It can be seen that on the surface of the NCO-5/1 electrode, after 200 charge/discharge cycles, a film of organic products of electrolyte decomposition was present and burned out by an electron beam (Figure S8). Considering SEM and CV investigations after a different number of cycles, it can be concluded that the current maximum at 1.6–1.7 V is related to SEI film, which was observed to grow during cycling.

To determine the influence of the chemical composition of the electrodes on the electrochemical activity, the shape of the anodic CV curves was analyzed (Figure 6c,d). The curves were normalized to the film thickness to allow for comparative analysis. With an increment of nickel oxide in films, a gradual shift in the maximum current intensity of the anodic curve from 2.04 V (CoO) to 2.23 V (NiO) and a change in the position of the maximum characterizing the SEI film from 1.6 to 1.7 V (Figure 6c) were observed. The shift towards high potentials of the maxima characterizing the electrochemical activity of the SEI film and nickel and cobalt oxides, with an increase in the proportion of nickel oxide, persisted even after 200 charge/discharge cycles (Figure 6d). After 200 charge/discharge cycles, the intensity of the maximum characterizing the SEI film’s electrochemical activity increased and became more significant than the total intensity of the maxima of nickel and cobalt oxides (Figure 6c,d).

Assuming that the increase of current observed on CV anode curves is proportional to electrochemical capacity and can be used to identify the contributions of the thin films’ components to the electrochemical performance during cycling, we subtracted the baseline and deconvoluted the anode curves. For NCO films, the anodic curves were simulated by Gaussian curves with maxima of 2.02 V (CoO) and 2.23 V (NiO). The peak maxima positions were determined based on the CV curves of pure oxides obtained in this work at a potential scan rate of 0.5 mV/s. The positions of maxima corresponding to the activity of CoO and NiO were fixed during modeling of NCO anode curves because they were retained even after cycling of CoO and NiO films during 200 cycles (Figure 6c,d). On the contrary, the position of the maximum revealing SEI activity was not fixed, since it changes during cycling. An example of Gaussian deconvolution for sample NCO-5/1 is shown in Figure 6b.

The change in the simulated Gaussian curve areas reflects the intensity of electrochemical processes in nickel oxide, cobalt oxide and SEI film, and characterizes the contribution of each component to the total electrochemical capacity (Figure 7).

The specific capacity in the initial charge/discharge cycles is mainly determined by the capacity of transition metal oxides (Ni, Co). The smallest SEI film formation was observed for the NCO-3/1 coating, where nearly 80% of the capacity was electrochemical processes involving nickel and cobalt oxides; for the other compositions, this figure was closer to 60% (Figure 7a).

After subtraction of SEI film capacity from the total, the following values of deposited transition metal oxide films were obtained: NiO—538 µAh/cm^2^/µm; NCO-5/1—581 µAh/cm^2^/µm; NCO-3/1—729 µAh/cm^2^/µm; NCO-1/1—539 µAh/cm^2^/µm; CoO—568 µAh/cm^2^/µm. The calculated values of TMO films at the initial cycles approached the theoretical values (Table 6).

After conducting 200 charge/discharge cycles, it can be seen that the area characterizing nickel oxide activity decreased slightly for all coating compositions. A more noticeable decrease in electrochemical activity was observed for cobalt oxide. The area under the curve characterizing the SEI film increased significantly for all considered coating compositions. The SEI film’s capacity became comparable to the capacity of TMOs (Figure 7b). For electrodes with monoxides (CoO and NiO) due to the formed SEI film, the largest increase in capacity was noted. For electrodes with the NCO-1/1 composition, a significant increase in capacity due to the SEI was also observed, presumably due to the different morphology of the films and the presence of whisker nanocrystals (Figure 4b). It should be noted that the contribution to the capacity of cobalt oxide decreased after cyclic tests for all compositions. Samples of the NCO-3/1 and NCO-5/1 series were characterized by the lowest SEI contribution to the total capacity.

Figure 7c shows the specific discharge capacities for the first cycles and after the cycle life test (200 cycles). Although the SEI film’s contribution increased, the overall capacity of CoO films decreased due to degradation of the TMO phase (Figure 7a,b). The NCO-3/1 and NCO-5/1 samples showed nearly equal capacity values before and after the cycle life tests. The growth of SEI film capacity compensated the loss of capacity due to transition metal degradation. The augmentation of NCO-1/1 and NiO total capacity (by a factor of 1.5–1.8) was related to SEI film growth caused by increased surface area (Figure 4b) and high concentration of nickel atoms at the surface, respectively.

## 4. Conclusions

Thin films of nickel-cobalt oxide were successfully prepared by ALD using nickelocene and cobaltocene as metal-precursors, and remote oxygen plasma as a counter-reagent. Changing the ratio of metal-precursor pulses allowed precise changing and controlling of the film composition. The films obtained possessed a uniform chemical composition along the depth with an admixture of metallic nickel and carbon up to 4 at.%. Comparing growth rate data of mono oxides (NO and CO) with the rates of NCO oxides showed that the growth of the phase or phases of the binary metals oxide was increased.

XRD data showed that NCO samples contained a spinel Ni_x_Co_(1−x)_O_2_-phase with a cubic structure Fm-3m. With increased Ni content of the Ni_x_Co_1−x_O phase, the unit cell parameter decreased from 4.244 to 4.161 Å. XRR data show that all samples consisted of a layer uniform in density with a roughness of 0.63 to 3.23 nm. With decreasing Ni content, the film density decreased from 6.60 to 6.31 g/cm^3^.

The SEM study of the chips of the film deposited on steel substrates revealed an increase in the GPC up to 35% compared to films deposited on the silicon substrates. On the surface of the NCO-1/1 samples with the smallest nickel content, nickel-oxide whisker nanocrystals were observed.

Electrochemical studies of all samples were carried out at charge current densities from 10 μA/cm^2^ (about 0.3 C) to 800 μA/cm^2^ (about 33 C). With an 80-fold increase in the current density, the samples’ discharge capacity decreased by only 21–33% (on average, from 920 to 655 µAh/cm^2^/µm). The moderate decrease in capacity was likely due to the small thickness of the films.

## Figures and Tables

**Table 1:** Conditions for atomic layer deposition (ALD) of Ni and Co oxides synthesis.

Deposited Oxides	Reagent	Co-Reactant	Pulse/Purge Times, Sec t_1_/t_2_/t_3_/t_4_	Reagent Temperature, °C	Reactor Temperature, °C	GPC Å/Cycle
NiO	NiCp_2_	O_2_ plasma	1/10/15/5	110	300	0.12
CoO	CoCp_2_			150		0.22

Note: t_1_—pulse time of metal-containing reagent, t_2_, t_4_—purge time of the reactor by nitrogen combined with evacuation, after reagent pulse of A and B, respectively, t_3_—surface treatment time with precursor B, GPC—growth per cycle.

**Table 2:** Transition metal oxide (TMO) films thickness and growth per cycle/supercycle measurement/calculation.

Sample	Ratio of Cycles		Number of Cycles/Supercycles	Ellipsometry, Å			XRR, Å			Calculation, Å		
	NiO	CoO		h	GPSC	GPC	h	GPSC	GPC	h	GPSC	GPC
NO	1	0	2308	272	-	0.12	280	-	0.12	-	-	-
NCO-5/1	5	1	310/1860	284	0.92	0.15	261	0.84	0.15	254	0.82	0.14
NCO-3/1	3	1	470/1880	338	0.72	0.18	300	0.64	0.16	273	0.58	0.15
NCO-1/1	1	1	900/1800	346	0.38	0.19	322	0.36	0.18	306	0.34	0.17
CO	0	1	500	110	-	0.22	174	-	0.34	-	-	-

Note: NO—NiO, NCO—Nickel-cobalt Oxide, CO—Cobalt Oxide, h—thickness.

**Table 3:** Chemical composition of NO, CO and NCO samples deposited on silicon supports based on X-ray photon electron spectra (XPS) data.

Sample	Sputtering Time, s	C	O	Ni	Co	Ni + Co-O	Ni/Co
		at.%					
NO	90	1.7	43.3	55.0	0	55.0	–
NCO-5/1	90	3.8	43.4	35.1	17.6	9.3	1.99
NCO-3/1	90	3.2	42.9	23.1	30.8	10.9	0.75
NCO-1/1	90	3.5	47.1	7.5	41.9	2.4	0.18
CO	45	3.9	45.3	0	50.7	5.3	–

**Table 4:** Unit cell parameters, density and roughness of the TMOs thin films.

Sample	*a*, Å	Density, g/cm^3^	Roughness, nm
NO	4.161	6.60	0.63
NCO-5/1	4.196	6.40	1.78
NCO-3/1	4.198	6.32	2.38
NCO-1/1	4.232	6.19	3.23
CO	4.245	6.31	1.12

**Table 5:** Film thickness on silicon and steel substrates, ratio, and the size of inclusions of studied TMO samples.

Sample	Thickness on Si, nm (Ellipsometry)	Thickness on Steel, nm (SEM)	Thickness on Steel/Thickness on Si	Inclusion Size on Si, nm	Inclusion Size on Steel, nm
NO	27	28–30	1.1	-	15–20
NCO-5/1	28	33–37	1.2	10–13	10–15
NCO-3/1	34	42–47	1.2	10–13	10–15
NCO-1/1	35	56–60	1.8	25–30	20–30
CO	11	32	2.9	-	25–35

**Table 6:** Specific capacities of TMOs.

Active Material	Density, g/cm^3^	Specific Capacity, mAh/g	Specific Volume Capacity µAh/cm^2^/µm	Current Density, mA/g|µA/cm^2^|C-Rate	Link Source
NiO	6.72	718	482.2	-	Theoretical (Faraday Law)
CoO	6.44	715	460.7	-	
NiCoO_2_	6.58 *	717	471.4	-	
NiO thin films	6.6	1460 981	967 647	32|20|0.3 2550|800|33	This Research
NCO-5/1 thin films	6.40	1430 1028	917 658	32|20|0.3 2550|800|33	
NCO-3/1 thin films	6.32	1442 1010	912 639	32|20|0.3 2550|800|33	
NCO-1/1 thin films	6.19	1440 1140.4	893 706	32|20|0.3 2550|800|33	
CoO thin films	6.31	1441.0 990	909 625	32|20|0.3 2550|800|33	
NiCoO_2_ Hierarchical mesoporous microspheres	6.58 *	845 397	555.7 261.0	90|-|- 4000|-|-	[7]
NiCoO_2_ nanotubes with Nanosheets	6.58 *	1130 300	743.5 197.4	200|-|- 800|-|-	[71]
NiCoO_2_@CNT1 Composites	6.58 *	1150 920	756.7 605.4	200|-|- 800|-|-	[71]
NCO_2_ carbon fiber nano-brushes	6.58 *	1250 300	822.5 197.4	200|-|- 2000|-|-	[72]
NiO–CoO nanosphere	6.58 *	1100	723.8	200|-|-	[72]
Mesoporous CoNiO_2_ hierarchical micro flowers	6.58 *	600 200	394.8 131.6	100|-|- 1000|-|-	[73]

Note: ^1^ CNT—carbon nanotubes; ^2^ NCO—NiCoO_2_, * the density of NiCoO_2_ was calculated based on NiO and CoO densities (Table 4).

## Data Availability

Data available on request due to restrictions on privacy. The data presented in this study are available on request from the corresponding author.

## Supplementary Materials

The following are available online at https://www.mdpi.com/article/10.3390/nano11040907/s1, Figure S1: NiO XRR pattern, Figure S2: NCO-5/1 XRR pattern, Figure S3: NCO-3/1 XRR pattern, Figure S4: NCO-1/1 XRR pattern, Figure S5: CoO XRR pattern, Figure S6: Cyclic voltammetry curves of NCO-1/1 sample in 0.08–3.00 V range, Figure S7: SEM image of NCO-5/1 thin film at 200 kX magnification, after CV (205 cycles), Figure S8: SEM image of NCO-5/1 thin film at 97.6 kX magnification, above figure 1 area after 24 s.
