# Supplementary material for: Atomic Layer Deposition of Ni-Co-O Thin-Film Electrodes for Solid-State LIBs and the Influence of Chemical Composition on Overcapacity
Source: Nanomaterials (Basel). 2021 Apr 2;11(4):907. doi: 10.3390/nano11040907 (PMC8065629; doi:10.3390/nano11040907)
Supplement: Supplementary file 1 [file nanomaterials-11-00907-s001.pdf]

*nanomaterials*

*Supporting Information*

# Atomic Layer Deposition of Ni-Co-O Thin-Film Electrodes for Solid-State LIBs and the Influence of Chemical Composition on Overcapacity

**Yury Koshtyal [1], Ilya Mitrofanov [1], Denis Nazarov [1,2], Oleg Medvedev [1], Artem Kim [1], Ilya Ezhov [1], Aleksander Rumyantsev [1,3], Anatoly Popovich [1] and Maxim Yu. Maximov [1,*]**

[1] Peter the Great Saint-Petersburg Polytechnic University, 195221 Saint Petersburg, Russia; yury.koshtyal@gmail.com (Y.K.); carlemeros@gmail.com (I.M.); dennazar1@ya.ru (D.N.); medvedev.os1990@gmail.com (O.M.); artem_7.kim@mail.ru (A.K.); iezhov1994@gmail.com (I.E.); rumyantsev.amr@gmail.com (A.R.); director@immet.spbstu.ru (A.P.)

[2] Saint Petersburg State University, 199034 Saint Petersburg, Russia

[3] Ioffe Institute, 194021 Saint Petersburg, Russia

* Correspondence: maximspbstu@mail.ru

**Discussion of nickel oxide nanowire/whiskers growth on the sample NCO-1/1**

There are several mechanisms of nanowire/whisker growth are presented in the literature, which can be used to describe the morphology of the NCO 1/1 sample. One of the currently widely used methods for the growth of nanowire/whisker is the vapor-liquid-solid (vapor-liquid-solid) method using nanocatalysts on the substrate surface [1–3], including for the growth of metal oxide whiskers [4]. Another way to obtain nanocrystals of transition metal oxides is thermal oxidation [5,6]. Thus, the work [5] emphasizes the importance of the composition of the NiCr bimetal for the formation of conditions for the growth of NiO whiskers during oxidation, and the temperature range is important, which determines the way copper oxide grows in the work [6].

Let us consider a possible model of the formation of nanowire/whisker on the surface of an NCO-1/1 sample and the effect of nickel and cobalt elements on the film growth process. An estimate of the density of whiskers on the NCO-1/1 sample and large crystallites on the CoO surface gives an identical value. Hence, it can be assumed that large CoO crystallites on the film surface can act as nano-catalysts for the growth of NiO whisker nanocrystals. However, the vapor-liquid-solid method is unlikely to be implemented, since the substrate temperature is 300C, which is insufficient for the formation of a liquid solution. It is known from the XPS results that in the sample NCO-1/1 the weight content of nickel is 1/6 of the mass of cobalt, while for the samples NCO-3/1 and NCO-5/1 the content of nickel atoms relative to cobalt is about 2/3 and 3/2 respectively. Apparently, the determining factor is the presence of a free surface with a given composition, which promotes the growth of a whisker [5]. Aligning the ratio of nickel and cobalt atoms in the film changes the conditions necessary for the formation of one-dimensional growth, which ultimately leads to a more uniform growth of the Ni-Co-O film without the formation of whiskers/needles. The construction of a more detailed model of the growth of on the NCO-1/1 sample requires additional experiments that are beyond the scope of this work.

The height of whiskers is close to the thickness of deposited film but not enough to cause any short circuits during cycling since the thickness of separator is much higher - 25 mm. However, in case of use of liquid electrolyte whiskers might serve as centers for transition metal deposition and their height might augment during cycling and cause internal short-circuit. For all-solid-state thin film batteries, the thickness of electrolyte layer (solid electrolyte - separator layer) is much thinner than in LIBs with liquid electrolyte. Thus, the presence of whiskers on anode leads to the necessity of formation a thicker solid-electrolyte layer to diminish the probability of its destruction and short circuit.

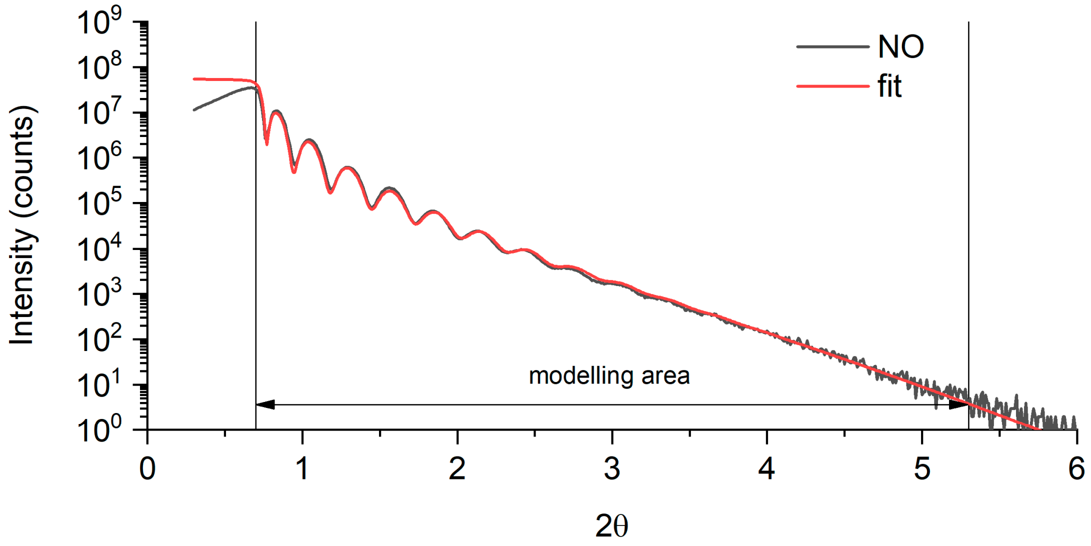


**Figure S1.** NiO XRR pattern.

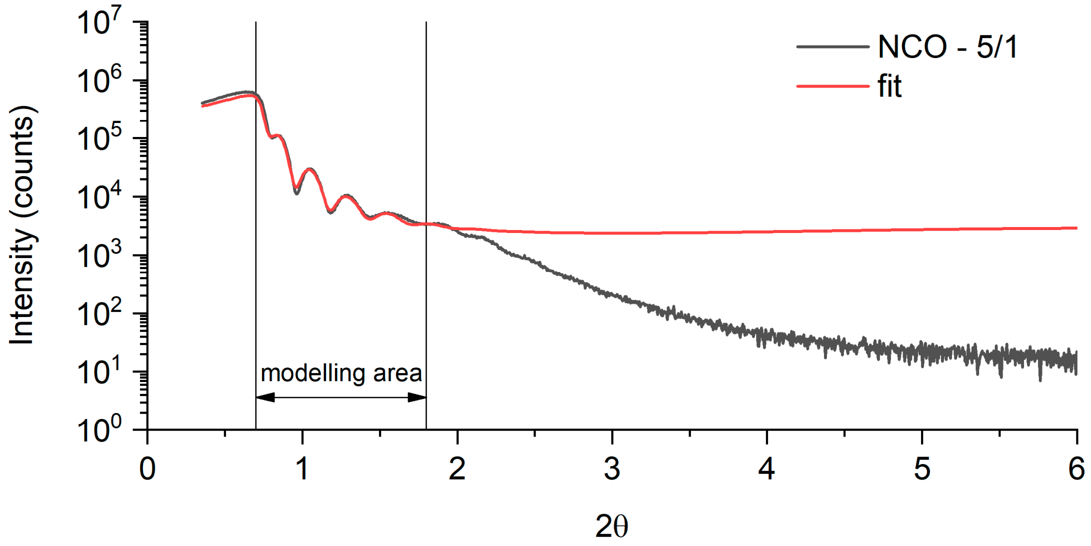


**Figure S2.** NCO-5/1 XRR pattern.

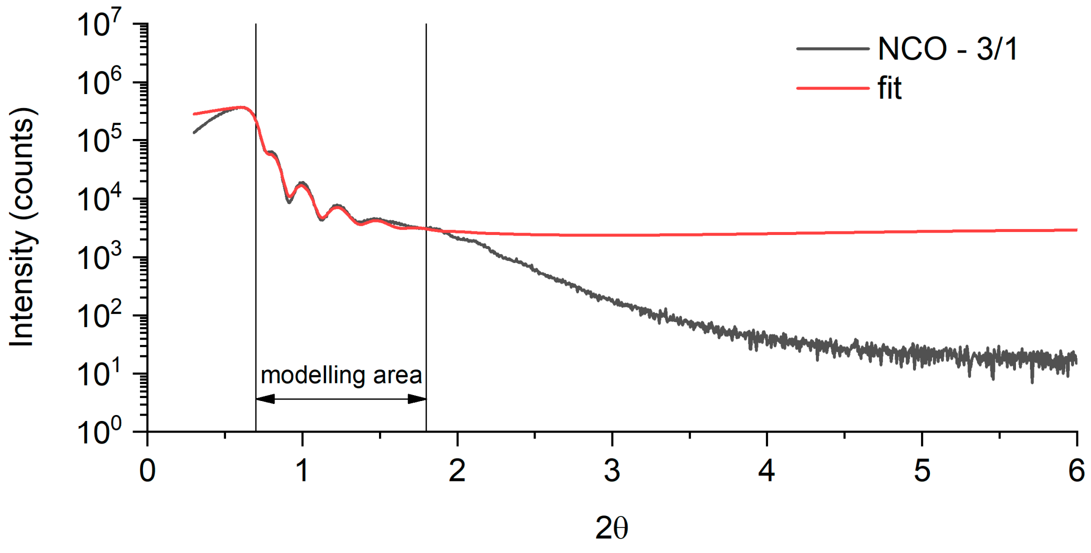


**Figure S3.** NCO-3/1 XRR pattern.

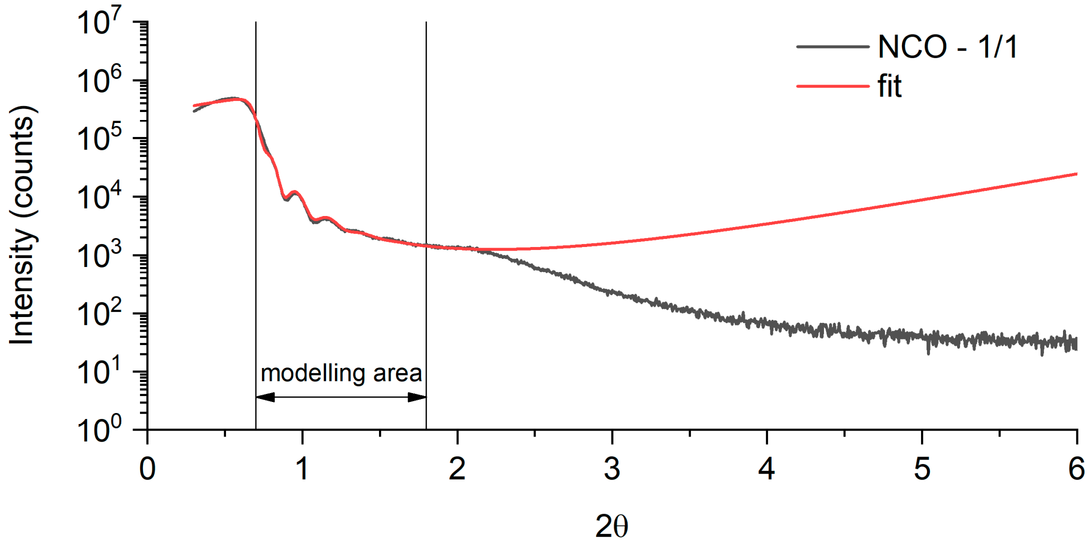


**Figure S4.** NCO-1/1 XRR pattern.

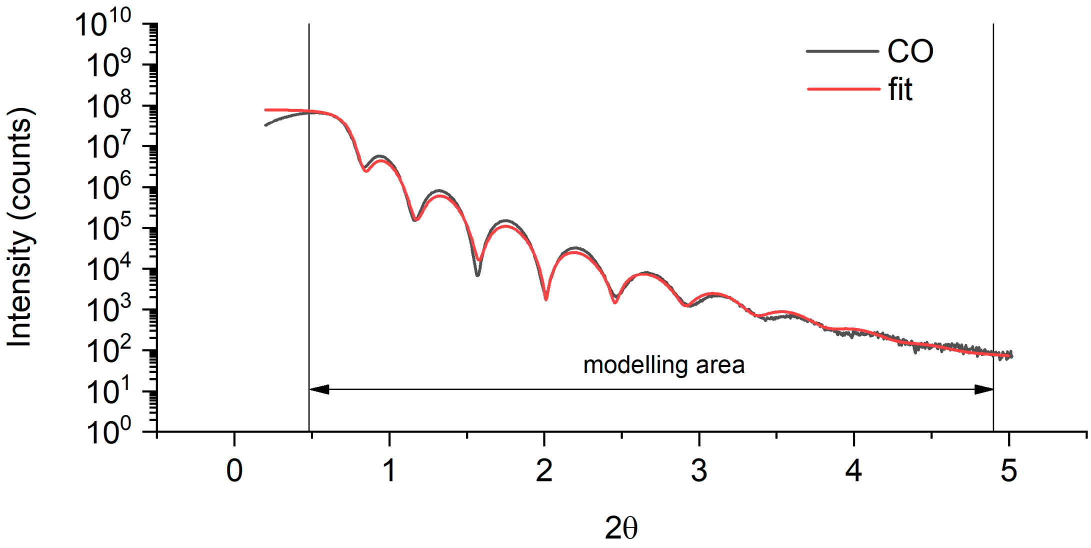


**Figure S5.** CoO XRR pattern.

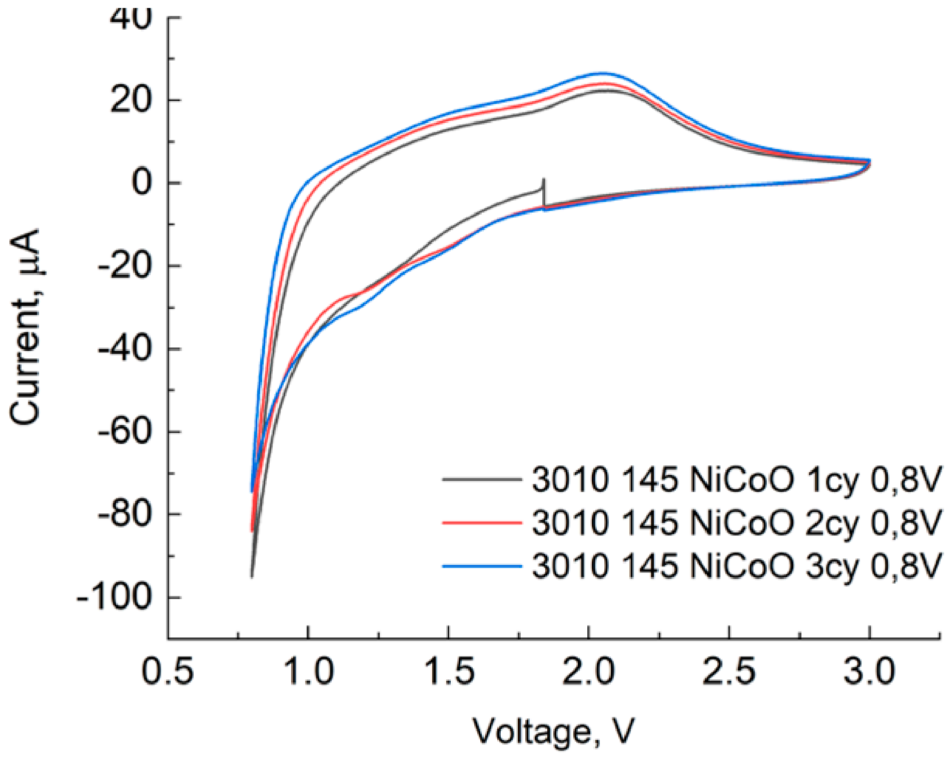


**Figure S6.** Cyclic voltammetry curves of NCO-1/1 sample in 0.08–3.00V range.

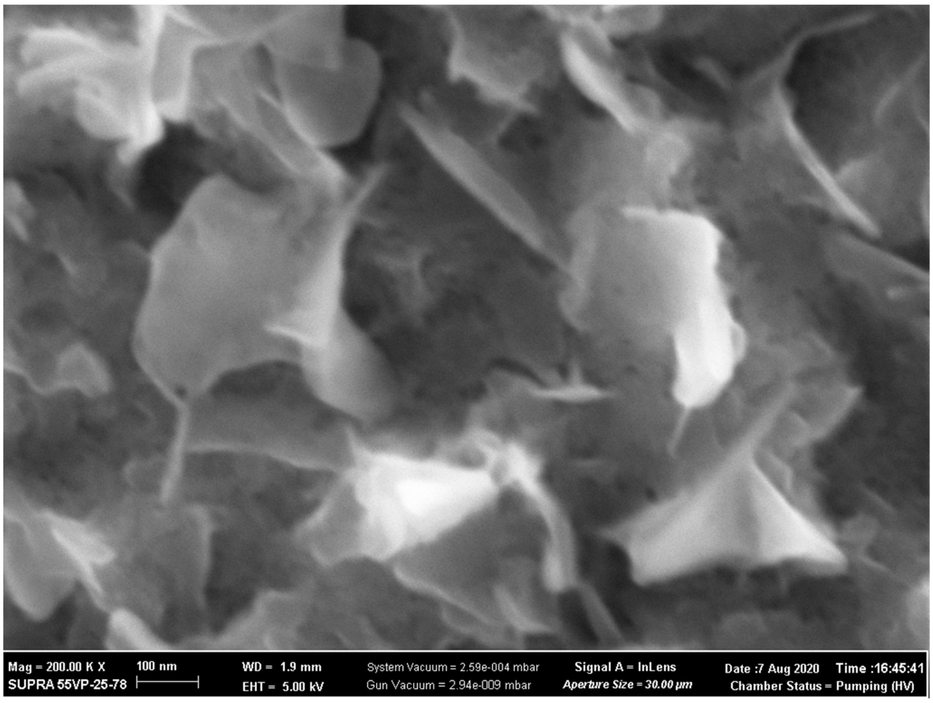


**Figure S7.** SEM image of NCO-5/1 thin film at 200 kX magnification, after CV (205 cycles).

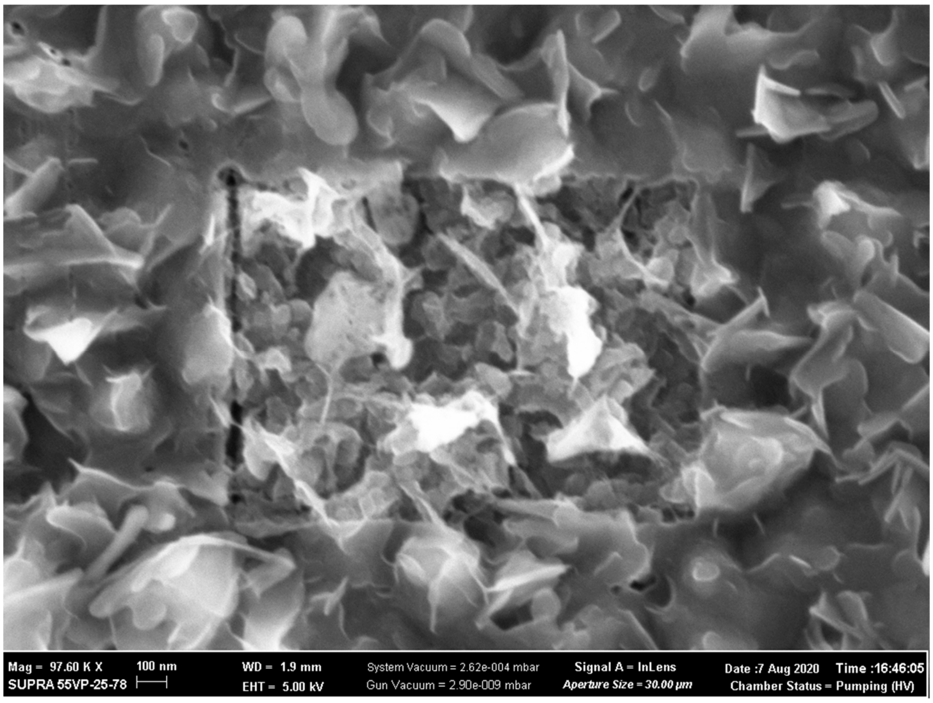


**Figure S8.** SEM image of NCO-5/1 thin film at 97.6 kX magnification, above figure 1 area after 24s.

## References

1. Adhikari, H.; Marshall, A.F.; Goldthorpe, I.A.; Chidsey, C.E.D.; McIntyre, P.C. Metastability of Au-Ge liquid nanocatalysts: Ge vapor-liquid-solid nanowire growth far below the bulk eutectic temperature. Acs Nano **2007**, *1*, 415–422, doi:10.1021/nn7001486.
2. Wang, T.; Jiang, T.; Meng, X. Gallium-assisted growth of InSb nanowire. Revista Mexicana De Fisica **2019**, *65*, 601–606, doi:10.31349/RevMexFis.65.601.

3. Dao, K.A.; Dao, D.K.; Nguyen, T.D.; Phan, A.T.; Do, H.M. The effects of Au surface diffusion to formation of Au droplets/clusters and nanowire growth on GaAs substrate using VLS method. Journal of Materials Science-Materials in Electronics **2012**, *23*, 2065–2074, doi:10.1007/s10854-012-0704-y.
4. Yu, H.K.; Lee, J.L. Growth mechanism of metal-oxide nanowires synthesized by electron beam evaporation: A self-catalytic vapor-liquid-solid process. Scientific Reports **2014**, *4*, 1–8, doi:10.1038/srep06589.
5. Luo, L.L.; Zou, L.F.; Schreiber, D.K.; Baer, D.R.; Bruemmer, S.M.; Zhou, G.W.; Wang, C.M. In-situ transmission electron microscopy study of surface oxidation for Ni-10Cr and Ni-20Cr alloys. Scripta Materialia **2016**, *114*, 129–132, doi:10.1016/j.scriptamat.2015.11.031.
6. Cao, F.; Jia, S.F.; Zheng, H.; Zhao, L.L.; Liu, H.H.; Li, L.; Zhao, L.G.; Hu, Y.M.; Gu, H.S.; Wang, J.B. Thermal-induced formation of domain structures in CuO nanomaterials. Physical Review Materials **2017**, *1*, 053401, doi:10.1103/PhysRevMaterials.1.053401.
